# Efficacy and tolerability of a 12-week combination chemotherapy followed by lomustine consolidation treatment in canine B- and T-cell lymphoma

**DOI:** 10.1186/s13028-022-00660-z

**Published:** 2022-12-12

**Authors:** Stefanie Limmer, Verena Nerschbach, Nina Eberle, Erik Teske, Daniela Simon Betz

**Affiliations:** 1 Small Animal Hospital, University of Veterinary Medicine Hannover, Bünteweg 9, Gebäude 280, 30559 Hannover, Germany; 2 Center for Small Animal Medicine, VetSpezial, Im Kornfeld 7, 31275 Lehrte, Germany; 3 Department of Clinical Sciences, Veterinary Faculty, Utrecht University, P.O. Box 80.154, 3508TD Utrecht, The Netherlands; 4 Independent Scientific Writing, Translation & Consultancy Clinical Oncology, Bünteweg 9, Gebäude 280, 30559 Hannover, Germany

**Keywords:** Dog, Prognostic factors, Remission, Short-term chemotherapy, Survival

## Abstract

**Background:**

High-grade lymphoma in dogs is a chemotherapy-responsive neoplasia with remission rates exceeding 80% under combination chemotherapy protocols. Usually these protocols are intensive and 24 + weeks. The objective of the present study was to investigate if a shorter protocol combined with an oral lomustine maintenance treatment (3 × in 8 weeks) would present an acceptable result, both for B- and T-cell lymphomas, and for the different types of lymphomas normally encountered in private veterinary practice.

**Results:**

144 dogs entered the study. Lymphoma types included multicentric (n = 123), alimentary (n = 13), miscellaneous (n = 7), and mediastinal lymphoma (n = 1). Overall response rate was 83.3% (B-cell: 86.6%, T-cell: 79.4%). Complete remission (CR) was achieved in 72.2% (B-cell: 77.3%, T-cell: 67.6%) and partial remission (PR) in 11.1% (B-cell: 9.3%, T-cell: 11.8%) of the dogs. Median duration of first CR amounted to 242 days (B-cell: 263 d, T-cell: 161 d). Median survival in dogs with CR was 374 days (B-cell: 436 d, T-cell: 252 d), and median overall survival time was 291 days (B-cell: 357d, T-cell: 210d). Immunophenotype demonstrated an independent significant influence on duration of remission and survival in the whole group. Findings of splenic and hepatic cytology were not significant associated with patient outcome. Treatment was well tolerated; the majority of adverse events were classified as grade 1 or 2.

**Conclusions:**

Short-term chemotherapy followed by lomustine consolidation leads to compara-ble remission and survival times compared to conventional protocols with cyclophosphamide, doxorubicin, vincristine and prednisolone with acceptable toxicosis in dogs with both B-cell and T-cell lymphoma.

**Supplementary Information:**

The online version contains supplementary material available at 10.1186/s13028-022-00660-z.

## Background

Lymphoma is one of the most frequently diagnosed neoplasms in dogs, with an estimated annual incidence of 13 up to 114 per 100,000 animals [1, 2]. A diversity of multi- and single-agent chemotherapy protocols with varying treatment durations have been described to treat this disease, leading to complete remission rates ranging up to 84% and median remission durations of around 300 days [3–9].

Recent studies showed that short-term protocols may represent an alternative to traditional long-term treatment regimens with a maintenance phase in that these yield a comparable response outcome [5, 7–10]. Several of these studies reported comparable first response duration [5, 7, 10, 11], while others reported shorter remission periods [9, 12]. Apart from reduced cost and higher owner acceptance, discontinuous chemotherapy protocols also bear the potential of reduced degree of toxicity and drug resistance [7, 10]. In addition, there is a benefit of the potential reduced use of (environment) hazardous drugs and improved work safety situation when reducing the amount of cytostatic agents handled and administered.

In dogs, lomustine traditionally has been used for treatment of resistant and relapsed lymphoma [12–16]. Lomustine has also been employed as first-line treatment in combination with prednisone or cyclophosphamide in a few clinical investigations [17, 18]. More recently, lomustine has found increasing acceptance, especially in treating canine high grade T-cell lymphoma [19, 20].

The vast majority of the chemotherapy protocols for the treatment of lymphoma include a combination chemotherapy protocol that can take 24 + weeks. For several owners this will present a logistic and financial problem. The aim of the present study was to investigate if a shorter protocol combined with an oral lomustine maintenance treatment (3 × in 8 weeks) would present an acceptable result, both for B- and T-cell lymphomas, and for the different types of lymphomas normally encountered in private veterinary practice. Therefore, response rates and response durations, as well as adverse events were used as primary objectives. Overall survival and prognostic factors were secondary objectives of this study.

## Methods

### Animals

Dogs with histologically or cytologically confirmed diagnosis of intermediate-/high-grade lymphoma were eligible for enrollment in this prospective study. Further eligibility criteria were the absence of serious medical illness limiting full compliance with the study, as well as signed owner consent. As the studied protocol was meant for standard situations in private practices steroid pretreatment was not an exclusion criteria. This study did not require official or institutional ethical approval. The animals were handled according to high ethical standards and national legislation.

### Staging

Staging was required for inclusion into the study and comprised physical examination, complete blood count (CBC), serum biochemistry, urinalysis, thoracic and abdominal radiographs, abdominal ultrasound, cytology of ultrasound guided fine needle aspiration biopsy of both liver and spleen, bone marrow aspiration cytology, an ECG and/or cardiac ultrasound in those animals in which history and physical examination gave rise for suspicion of an abnormality and in breeds that are predisposed for cardiomyopathy. Tumor measurements were performed either directly or via radiographic or sonographic imaging. Clinical staging was performed according to the WHO clinical staging system for lymphoma in domestic animals [21]. Clinical signs that could be associated with the tumour like weight loss, gastro-intestinal disorders, polyuria-polydipsia, respiratory distress and fever were used for substaging into a and b. Immunophenotyping of the lymphomas was performed via flowcytometry analysis (markers: anti-CD21, anti-CD79, anti-CD3, anti-CD3 epsilon) of material aspirated from lymph nodes as reported before [22].

### Chemotherapy regimen and patient monitoring

Dogs were treated with a 12-week cyclic combination chemotherapy protocol consisting of L-asparaginase^1^, vincristine^2^, cyclophosphamide^3^, doxorubicin^4^ and prednisolone^5^ (Table 1). Dexamethasone^6^ or prednisolone^7^ was given directly before the administration of L-asparaginase and doxorubicin. Subsequently, lomustine consolidation treatment was performed only in dogs that had achieved a complete remission (CR) in week 14, 17 and 20.

**Table 1:** Multi-agent short-term chemotherapy protocol used for the treatment of lymphoma in 144 dogs

		Week																			
Substance	Dose	1	2	3	4	5	6	7	8	9	10	11	12	13	14	15	16	17	18	19	20
L-asparaginase	400 IU/kg SC	•																			
Vincristine	0,7 mg/m^2^ IV	•			•			•			•										
Cyclophosphamide	250 mg/m^2^ PO/IV		•			•			•			•									
Doxorubicin^a^	30 mg/m^2^ IV*			•			•			•			•								
Lomustine^b^	70 mg/m^2^ PO														•			•			•
Prednisolone	40 mg/m^2^ PO × 3 days 30 mg/m^2^ PO × 3 days	•																			
	20 mg/m^2^ PO × 3 days 10 mg/m^2^ PO × 3 days		•																		

^a^Prior treatment with dexamethasone or prednisolone; dogs < 15 kg: 1 mg/kg

^b^Only if CR after 12 weeks

IV* intravenous infusion over 30 min

•, drug administered

Prior to each treatment a CBC was performed. ECG and echocardiography as seen indicated by the attending clinician were performed prior to each doxorubicin treatment. Serum biochemistry was performed when any clinical sign of systemic toxicosis occurred. Furthermore, bile acids (and in case of increased bile acids and / or signs of liver toxicity full hepatic enzymes profile) were analyzed prior to each lomustine administration. Tumor size was evaluated at each visit during physical examination or if deemed necessary by the veterinarian by ultrasound. In case of CR, treatment was terminated after the 3^rd^ lomustine treatment and dogs were re-evaluated at 4-week intervals. Dogs not reaching CR were continued on cyclophosphamide, doxorubicin, vincristine and prednisolone (CHOP)-based chemotherapy depending on the owners’ choice. Post-treatment monitoring was conducted by re-check examinations by the attending clinicians every 6 weeks for 6 months after the completion of the protocol followed by every 3 months onwards. In case of relapse, dogs were treated with a second line chemotherapy protocol, either with the initial protocol or one of different rescue protocols, subject to the owner’s decision.

### Response assessment

Responses (CR, partial remission (PR), stable disease (SD), progressive disease (PD)) and response durations were classified using RECIST criteria according to the Veterinary Co-Operative Oncology Group (VCOG) consensus statement for lymphoma [23]. Responses were assessed in an intent-to-treat analysis.

### Assessment of toxicity

Results of CBC taken one week after each treatment, as well as signs of gastrointestinal or any other toxicity noticed by owners or clinician were recorded. Adverse events were graded according to the VCOG’s common terminology criteria for adverse events [24]. Number of neutropenic, anemic and thrombocytopenic episodes as well as episodes of gastrointestinal (anorexia, diarrhea, vomiting) or any other toxicity (e.g. lethargy, urocystitis) throughout the treatment and furthermore the associated drugs were documented for each patient visit.

### Statistical analysis

CR and PR rates were defined as number of dogs achieving complete or partial remission compared to the total number of treated dogs. Overall survival (OS) was defined as time from induction treatment to death or the last date of the dog known to be alive. Disease free interval (DFI) was calculated for those dogs which went into CR as the interval between moment of confirmation of CR and relapse or the date that the dog was last known to be free of disease, only counting relapses as events. Overall remission duration was defined as time from (complete or partial) remission to relapse or death.

Survival curves were calculated with the Kaplan–Meier product limit analysis. Differences in survival between groups were determined with the log-rank test. Dogs were censored for survival and remission analysis when they were alive at the time of data accrual closure, dead due to causes other than lymphoma or lost to follow up and were still in remission at time of death or data accrual closure, respectively.

Univariate Cox regression analysis, followed by multivariate Cox backward regression analysis including all parameters with a P-value < 0.15 in univariate analysis, was performed to evaluate the following variables for their independent influence on remission and survival times: age, body weight, gender, neuter status, anatomic classification, clinical stage, substage, laboratory parameters at time of diagnosis (numbers of leukocytes, lymphocytes, and neutrophils), presence of anemia, thrombocytopenia or hypercalcemia at diagnosis, immunophenotype, results of radiologic and cytological examination of spleen and liver.

Normality of continuous data was tested by the Kolmogorov–Smirnov test. Based on this, non-parametric tests were used for significance testing.

A P-value of < 0.05 was considered significant. All statistical analyses were performed using IBM SPSS Statistics 26 software^g^.

## Results

### Patient population

One hundred and forty-four dogs entered the study between 2004 and 2011 (Additional file 1). Breeds represented were mixed-breed (n = 40), Golden retriever (n = 10), Bernese mountain dog (n = 10), German shepherd (n = 8), Beagle (n = 6), Cocker spaniel (n = 6), Rottweiler (n = 5), Hovawart (n = 4) and the remaining 55 dogs were of 32 other breeds. Median age was 7.3 years (range, 3–16), median body weight was 30.3 kg (range, 5–59). Gender distribution was as follows: 81 dogs were male, (male castrated, n = 22); 63 dogs were female, (female spayed, n = 31). Pretreatment with corticosteroids was recorded for 47 dogs (32.6%), in an additional 7 dogs corticosteroid pretreatment was questionable, whereas the remaining 90 dogs (62.5%) had received no prior treatment. As no reliable data on the duration of corticosteroid pretreatment could be obtained in a large number of cases, this was not evaluated further. Median duration of clinical signs at diagnosis amounted to 21 days (range, 0–180).

### Clinical staging

Multicentric lymphoma was diagnosed in 123 dogs (85.4%), alimentary lymphoma in 13 dogs (9%), miscellaneous lymphoma in 7 dogs (4.9%), and the remaining dog had mediastinal lymphoma (0.7%). In 84.7% of the dogs (n = 122) diagnosis was based on cytology, in 1.4% (n = 2) on histopathology, and in 13.9% (n = 20) both investigations were performed. Cytology of fine needle aspirations of liver and spleen was performed in all dogs and revealed lymphoma involvement in 96 and 114 dogs, respectively. Bone marrow aspiration cytology could be analyzed in 130 dogs, showing lymphoma involvement in 43 animals (30%). One individual was graded as clinical stage II (0.7%) and 5 dogs as stage III (3.5%), the majority of the dogs were classified as clinical stage IV (n = 70, 48.6%) and stage V (n = 68, 47.2%). Sixty-one dogs (42.4%) were substage a and 83 dogs (57.6%) substage b.

### Laboratory parameters and immunophenotype

At the time of diagnosis 58 animals (40.3%) were anemic with hematocrit (HCT) ranging from 14 to 39% (median, 35%). Considering all dogs, median HCT was 41% (range, 14–61). Thrombocytopenia at initial presentation was detected in 32 dogs (22.2%) with platelet counts ranging from 24,000–148,000 cells/µL (median, 100,500 cells/µL, normal range, > 150,000 cells/µL). Median number of thromboyctes/µL in all dogs amounted to 247,000. Neutropenia was evident in 3 dogs (2.1%; range, 1320–1970 cells/µL, normal range, > 2000 cells/µL). In 16 dogs (11%), hypercalcemia was apparent (ionized calcium concentration 1.50–2.26 mmol/L; median, 1.78 mmol/L, normal range, < 1.47 mmol/L).

Immunophenotyping revealed B-cell lymphoma in 97 dogs (67.3%) and T-cell lymphoma in 34 (23.6%), in the remaining dogs phenotyping was not technically possible (n = 8; 5.6%) or yielded unclear results (n = 5; 3.5%).

B-cell immunophenotype of the lymphoma was more often associated with lymphoma involvement of spleen (P < 0.001) and liver (P = 0.001) than T-cell lymphoma. T-cell immunophenotype was more often associated with substage b (P = 0.011). All dogs with hypercalcemia tested for immunophenotype (14/16) had a T-cell lymphoma.

### Treatment outcome

Median time from diagnosis to first chemotherapy amounted to 3 days (range 0–39 days). Median time from first treatment to documentation of remission was 10 days (range 2–92 days). Response to treatment in the complete group of 144 dogs was as follows: CR: 104 (72.2%), PR: 16 (11.1%), SD: 9 (6.3%), and PD: 15 (10.4%) (Table 2). Between dogs achieving a CR (n = 104) and those not achieving a CR (n = 40) significant differences were evident regarding the following parameters: age, hematocrit, anemia, anatomic subtype, substage, and size of the spleen on radiographs (Table 3).

**Table 2:** Summary of treatment outcome

	All dogs (n-144)	Dogs with B-cell lymphoma (n = 97)	Dogs with T-cell lymphoma (n = 34)
Response to treatment			
CR	104 (72.2%)	75 (77.3%)	23 (67.6%)
PR	16 (11.1%)	9 ( 9.3%)	4 (11.8%)
SD + PD	24 (16.7%)	13 (13.4%)	7 (20.6%)
Median remission duration for			
CR (= DFI)	242d	263d	161d
CR + PR	240d	253d	161d
Median survival duration for			
All dogs	291d	357d	210d
Dogs with CR	374d	436d	252d

*CR*  complete remission, *PR* partial remission, *SD* stable disease, *DFI* disease free interval

**Table 3:** Possible prognostic factors predicting probability for complete remission in 144 dogs with lymphoma

Variable	P-value	Higher CR probability with
Age (years)	0.004	Lower age
Gender (M/F)	0.822	
Neutering (Y/N)	0.818	
Weight (kg)	0.159	
Corticosteroid pretreatment (Y/N)	0.853	
Anatomical subtype	0.026	Multicentric lymphoma
Stage (1–5)	0.845	
Substage	0.023	Substage a
Immunophenotype (B/T)	0.405	
Anaemia (Y/N)	0.004	No anaemia
Hematology		
Hematocrit (l/l/)	0.026	Higher hematocrit
Lymphocytes number	0.351	
Neutrophils number	0.101	
Platelets number	0.281	
Hypercalcemia (Y/N)	0.895	
Ultrasound spleen	0.448	
Ultrasound liver	0.691	
Xray size spleen (1–3)	0.008	No enlargement
Xray size liver (1–3)	0.341	
Cytological involvement spleen (Y/N)	0.120	
Cytological involvement liver (Y/N)	0.227	

### Remission and survival duration analysis

At the time of data accrual closure 18 dogs were alive, 122 had died (100 due to lymphoma) and 4 dogs were lost to follow up.

**Fig. 1:**
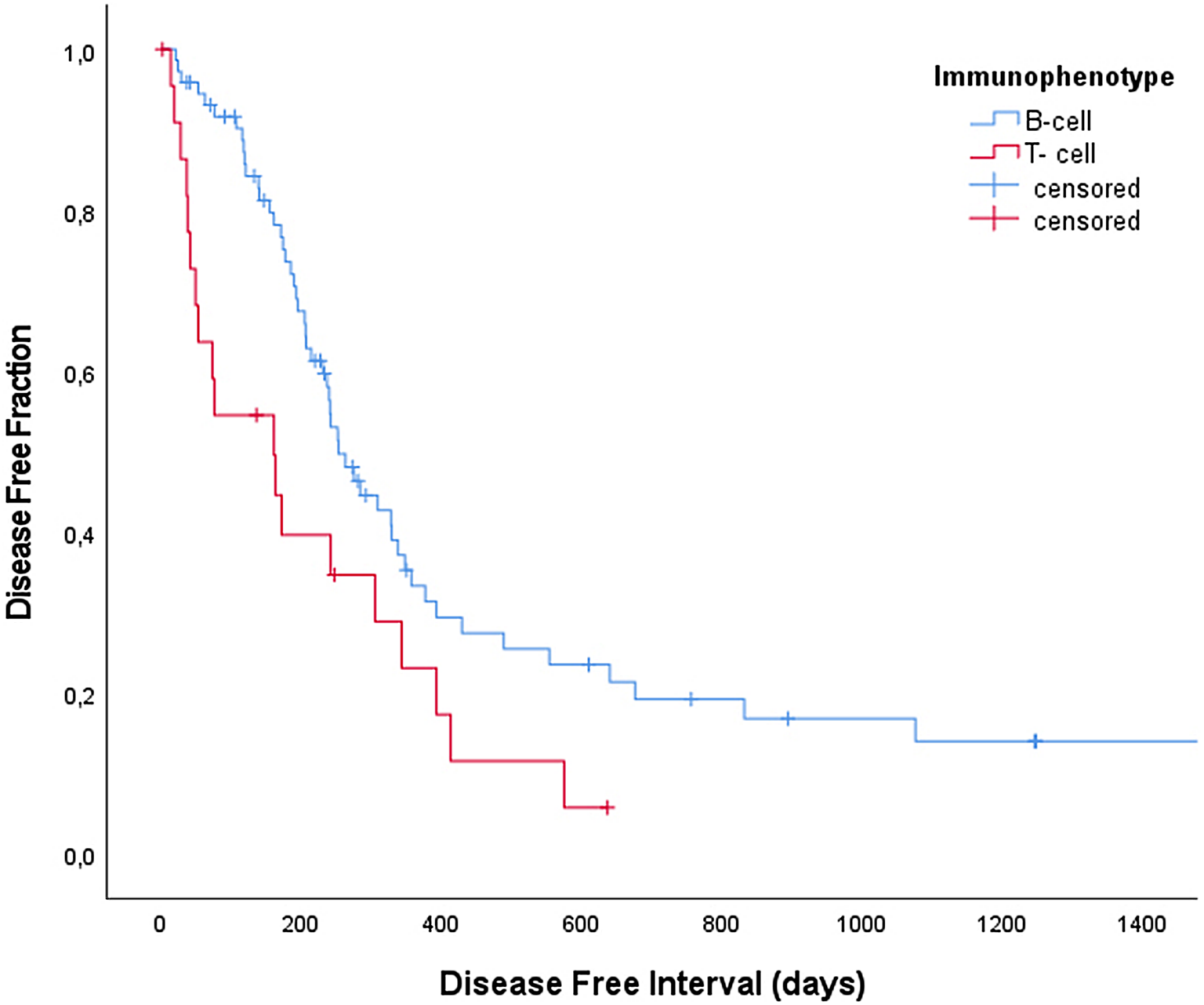
Kaplan–Meier curves of duration of DFI in dogs with B-cell lymphoma (blue line, n = 75) and T-cell lymphoma (red line, n = 23) reaching complete remission following treatment with a 12-week CHOP-based protocol complemented by lomustine consolidation (P = 0.013)

Median Disease Free Interval (DFI), calculated for dogs with a CR only, amounted to 242 days (n = 104, range, 2–2034 days), with 1- and 2-year DFI rates of 30.3% and 16.1%, respectively. Median overall remission duration (for CR and PR) was 240 days (n = 120, range 0–2034 days), with 1- and 2-year remission rates of 29.8% and 16.8%, respectively. In both groups of dogs (CR only and CR + PR), multivariate Cox regression analysis showed that the variable immunophenotype retained independent influence on DFI and overall remission duration (*P* = 0.021 and P = 0.015, Fig. 1). Median DFI of B-cell lymphoma amounted to 263 days (range: 21–2034 days), with 1- and 2-year remission rates of 32% and 20%, respectively. Median DFI of T-cell lymphoma amounted to 161 days (range: 2–637 days), with 1- and 2-year remission rates of 23% and 0%, respectively.

**Fig. 2:**
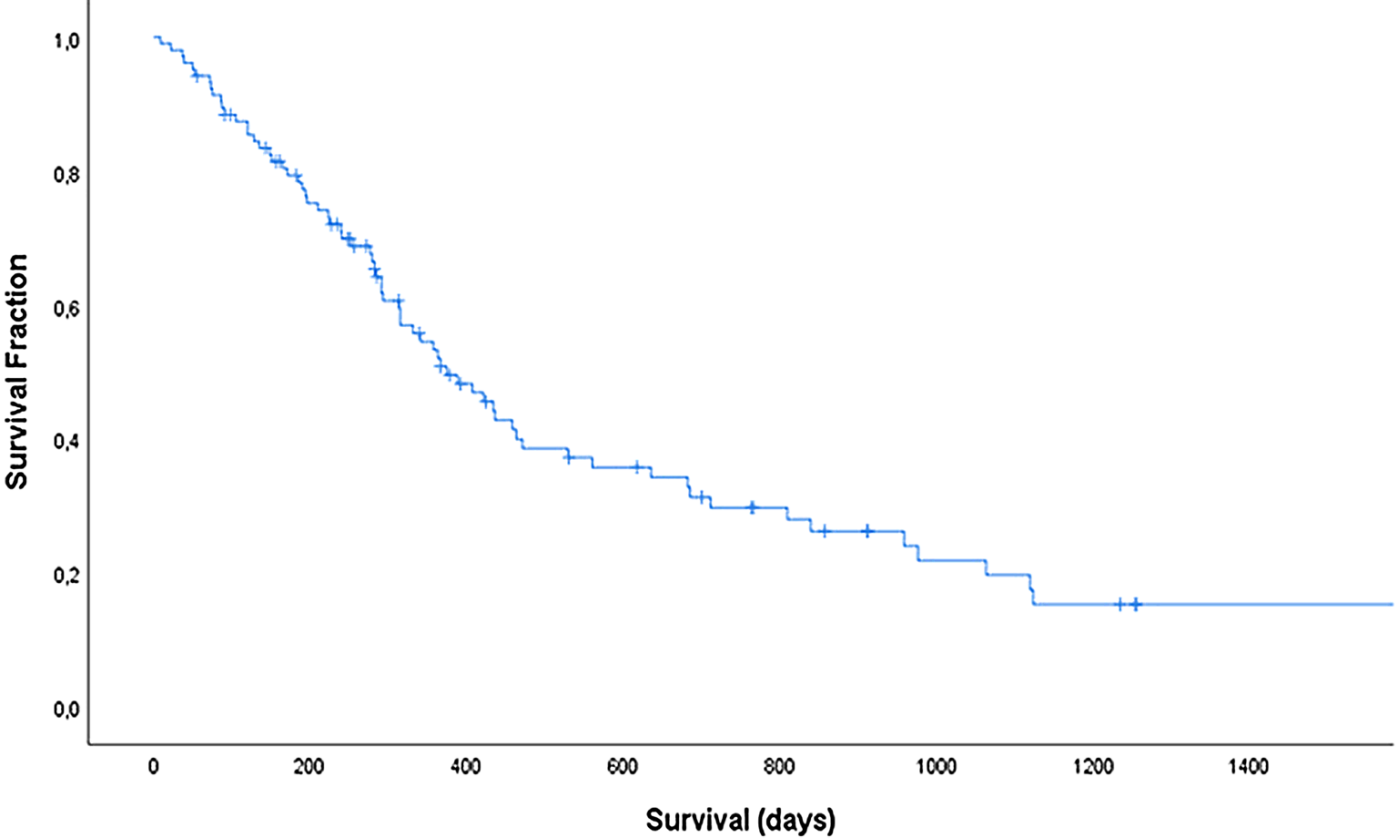
Kaplan–Meier curve depicting survival duration for dogs reaching complete remission following treatment with a 12-week CHOP-based protocol complemented by lomustine consolidation

Median overall survival duration amounted to 291 days (n = 144, range 2–1123 days), with 1- and 2-year survival rates of 41% and 25%, respectively (Fig. 2), whereas median survival of dogs achieving a CR was 374 days (n = 104, range 9–1123 days), with 1-and 2-year survival rates of 52% and 30%, respectively.

**Fig. 3:**
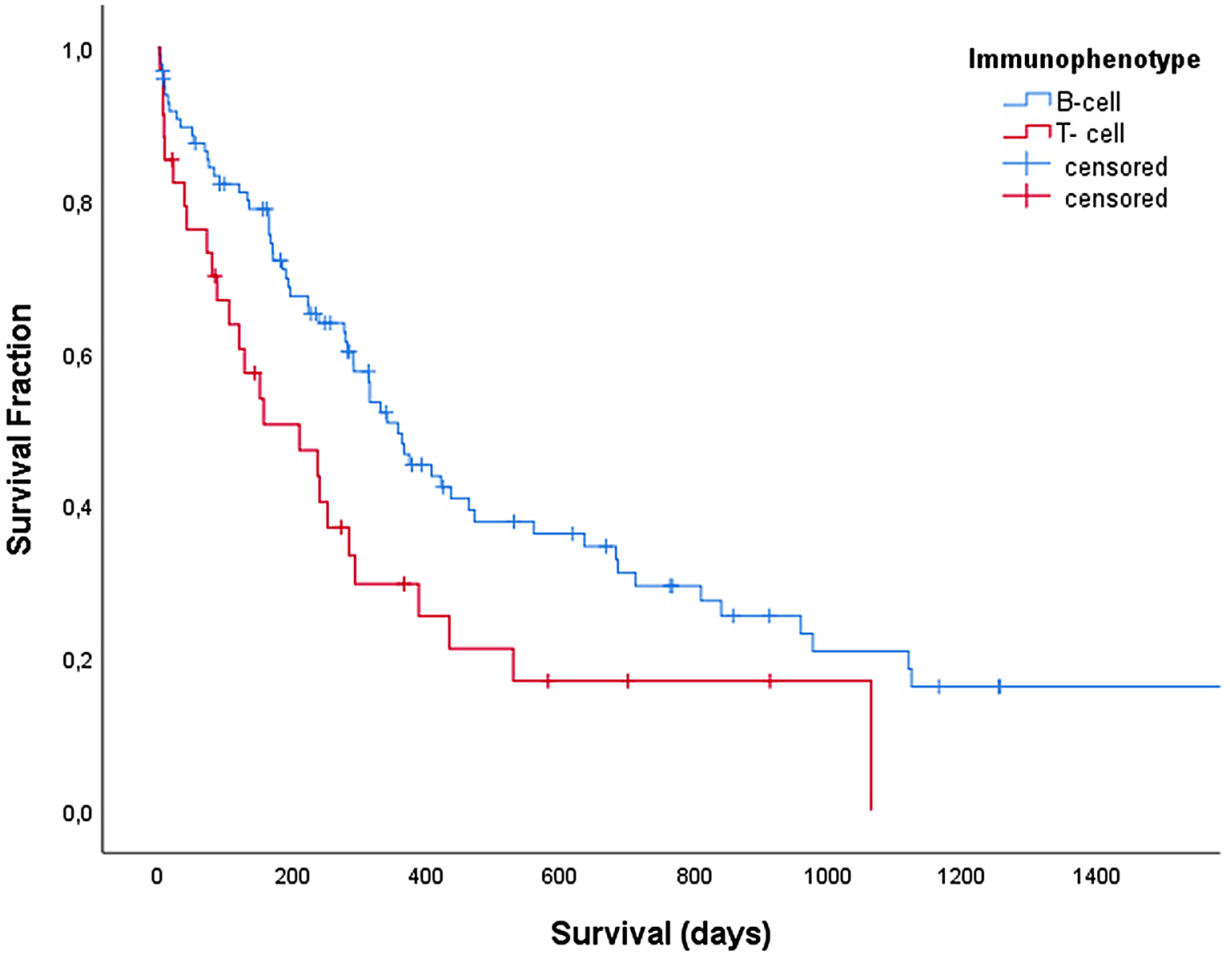
Kaplan–Meier curve depicting overall survival for dogs with B-cell (blue line, n = 97) and T-cell lymphoma (red line, n = 34) treated with a 12-week CHOP-based protocol complemented by lomustine consolidation

When applying multiple regression analysis a significant model (P = 0.003) was built, which indicated that pre-treatment with corticosteroids, a higher absolute neutrophil count, presence of anaemia or thrombocytopenia and T-cell immunophenotype were associated with a shorter survival (Fig. 3).

### Remission after relapse

Of the 104 dogs reaching complete remission, a relapse developed in 74 cases. Fifty-eight dogs received subsequent chemotherapy, 43 of which being treated according to the same CHOP protocol. Second response rates in these dogs were CR 27/43 (62.8%), PR 8/43 (18.6%), SD 2/43 (4.7%) and PD 6/43 (14%).

### Adverse events

The number of neutropenic episodes occurring in each dog during the 20-week treatment time ranged from 0 to 7 (median: 1), 35% (n = 51) of the dogs had no neutropenic episode (Additional file 2). Of the dogs experiencing neutropenia (n = 85), a single neutropenic episode was found in 46 animals, 2 and 3 episodes in 12 animals each, 4, 5 and 6 episodes in 5, 6 and 3 animals, respectively and 1 individual underwent 7 episodes of neutropenia. A total number of 181 neutropenic episodes were documented in the study population, 45% of these cases was neutropenia grade 1 (n = 82), 30% grade 2 (n = 55), 18% grade 3 (n = 33), and 6% grade 4 (n = 11) of cases. Forty-four (24%) neutropenic episodes occurred after the first treatment with vincristine and L-asparaginase. Distribution of the remaining episodes of neutropenia was: 41 (23%) after treatment with vincristine, 38 (21%) after treatment with cyclophosphamide, 43 (24%) after treatment with doxorubicin and 15 (8%) following lomustine administration.

In total, 173 treatment delays in 83 dogs (65%) (median 1, range, 0–5) were documented, 77% (n = 134, in 67 dogs) of these postponements were caused by neutropenia. The number of neutropenic events was not correlated with DFI (P = 0.804) nor with OS (P = 0.130).

Additional hematologic toxicoses (anemia, thrombocytopenia) as well as lethargy were documented for each dog and were mostly grade 1 and grade 2.

Bile acid abnormalities were not recorded before or during the lomustine treatment cycles, therefore full liver enzyme profiles were not analyzed.

Gastrointestinal toxicosis was reported as anorexia, diarrhea or vomiting. Episodes of anorexia occurred 111 times in 70 dogs (51%) with 49% (n = 54) being grade 1 and 42% (n = 47) grade 2. Diarrhea and vomiting appeared 87 and 104 times in 38% (n = 55) and 42% (n = 60) of the dogs, respectively, and were mostly grade 1 and 2 as well.

Clinical hemorrhagic urocystitis was documented 13 times in 13 dogs (9%), of which 9 were reported directly after cyclophosphamide was given, and 4 after a few weeks. When hemorrhagic cystitis occurred cyclophosphamide was replaced by chlorambucil.

Dogs achieving a CR had a significantly higher number of anemic episodes than both dogs achieving a PR (P = 0.011) or a PD (P < 0.001), and dogs with a CR or PR experienced more frequent episodes of diarrhea than dogs with a SD (P = 0.016 and P = 0.043, respectively). Vomiting episodes occurred in higher frequency in dogs with a CR than dogs with a SD (P = 0.049), while cystitis episodes showed no relationship to remission status.

## Discussion

Treatment of canine lymphoma has undergone a significant development in the past decades. Initially, long-term chemotherapy protocols combining different agents in an induction period as well as a long-lasting maintenance phase were implemented [25–27]. In recent years, a progression towards short-term combination protocols has occurred as these were shown to yield similar results as their long-term counterparts [4–7, 10, 28]. Looking at the main objectives of response rates and DFI, the presented discontinuous protocol with its 12-week combination phase complemented by three cycles of lomustine consolidation supports these observations in that the attained efficacy is well comparable to the prior maintenance-free regimes as well as long-term therapies. Comparison to prior studies is subject to a number of limitations, however taking these into account, the outcome results of the presented study seem well in line with those of other discontinuous CHOP-based trials with CR rates approximating around 80%, median CR durations/DFI ranging around 200–300 days, and median overall survival ranging around 260–310 days [6, 7, 9, 29, 30]. Other studies have demonstrated before that these shorter protocols have comparable responses rates, DFI and overall survival than longer procols with a maintenance phase [3, 5–9, 11, 25–30, 32].

Thus, encompassing a total treatment time of 20 weeks, this protocol is among the shortest of the currently described discontinuous regimes investigated in canine lymphoma. In this, a further clinical study underlines the clinical assumption that more and longer chemotherapy administration does not necessarily lead to improvement of remission rates and survival times. It is important to state that the value of the secondary end point overall survival is limited by the fact that it is strongly dependent on the owner’s decision concerning timing of euthanasia or choosing to pursue rescue treatment, the latter also possibly being subject to financial constraints. This aspect, however, is not unique to this investigation and a source of error found across veterinary clinical studies.

Following the important paradigm shift from implicating long-term—sometime life-long—chemotherapy for lymphoma dogs on to the acceptance of discontinuous treatments allowing for chemotherapy-free periods of variable duration, veterinary oncology is facing a further major transformation in how we define and treat canine lymphoma. It is now widely accepted that lymphoma does not represent a single disease entity but summarizes a diverse group of neoplasms with varying biologic behaviors and prognoses [31].

Already early-on, the differentiation between B- and T-cell immunophenotype was shown to possess prognostic value [30] and this remains to be the most consistent determinant of outcome supported by numerous clinical studies [4, 7, 30, 33–35]. The presented study again mirrors this important finding, with overall and CR remission durations as well as survival times displaying statistically significant differences independent of other variables between the B- and T-cell lymphomas.

Based on this and considering the still unsatisfactory outcome achievements in dogs with T-cell lymphoma, B- and T-cell lymphoma have been investigated as separate entities in more recent studies concerning both their population characteristics as well as the pertaining response to treatment and associated prognoses.

In the presented population of dogs with B-cell lymphoma, the outcome results are well in accordance with other reports in the literature: Two recent retrospective investigations in dogs with large B-cell lymphoma describe CR rates of 71.2% and 81.6% as well as median times to progression of 180 days and 252 days, respectively, following CHOP-based chemotherapy as well as variable other protocols [36, 37].

Even more importantly, considering their comparably less favorable prognosis the characterization of populations with T-cell lymphoma as an entity on its own is gaining more attention. Increasing evidence is apparent that especially dogs with T-cell-lymphoma may benefit from an incorporation of lomustine into the treatment plan. This was in particular demonstrated by a clinical trial using the combination of lomustine with vincristine, procarbazine, and prednisone (modified ‘LOPP’) as first-line treatment, resulting in a CR rate of 83%, as well as progression-free and overall survival of over 430 and 500 days, respectively [35]. Two further studies investigating a LOPP-type regime also report high response rates and overall survival times over 320 days [19, 20]. In addition, investigations separately evaluating T-cell-lymphoma populations treated without lomustine describe substantially lower overall survival times ranging between 120 and 270 days [4, 34, 38, 39].

Taking the limitations of inter-investigational comparisons into account, the outcome in the presented dogs with T-cell lymphoma seems comparable to the classical CHOP-based protocols in the studies mentioned above. However, the response rate, as well as overall remission and survival durations range well below those of the cited modified LOPP study [35]. The difference is the timing of lomustine in the protocol. As addition of lomustine appears to be beneficial in the treatment of T-cell lymphomas the timing of administration could be crucial. Therefore, a consolidation with lomustine at the end of the treatment regime would not provide the optimal benefit, as a varying number of T-cell lymphoma dogs may not even reach this part of the protocol. Future studies could hence focus on instituting lomustine early on, thus enabling each dog within the T-cell lymphoma study population to receive the agent in addition to the additional chemotherapy cycles. The encouraging remission and survival durations of the presented T-cell lymphoma dogs that obtained a complete remission and were able to reach the lomustine treatment cycles probably underline the importance of further investigations of timing of inclusion of this drug into the protocol.

Interestingly, the variables that apart from immunophenotype displayed independent influence on duration of remission and survival times were higher numbers of neutrophils and presence of anemia at diagnosis, respectively. This is also in line with previous recent studies showing that especially the leukocyte or neutrophil numbers in dogs with lymphoma may carry prognostic value [30, 36, 37, 40, 41]. The presence of anaemia has been reported before as a prognostic indicator [37]. The pathogenesis contributing to anaemia in these dogs is still undetermined and could be multifactorial. With hematology being an essential and non-invasive part of every staging process, results may provide useful prognostic information. Already since several decades pretreating dogs with corticosteroids have been associated with a poorer treatment outcome [3]. Also in our study pretreatment with corticosteroids was a negative prognostic factor for overall survival, but not for DFI. It is unclear why this is the case. If steroids will influence the efficacy of the chemotherapy protocol, it should also have an influence on the DFI. It has been postulated that pretreatment with corticosteroids will lead to drug resistance, however, corticosteroid treatment could not been associated with an increased ABC transporter expression causing this drug resistance [42].

A further interesting aspect to mention actually concerns the variables *not* displaying significant association with outcome. In the presented study population as a whole and within the B-and T-cell subgroups, splenic and hepatic cytology findings failed to prove any prognostic value for the remission or survival duration. The observation that whether spleen or liver are cytologically lymphoma-cell positive or negative has no impact on the patient’s outcome is in accordance with a previous recent publication demonstrating the identical result [43]. Hence, this in the least questions the utility of performing ultrasound-guided fine-needle aspirations as routine constituent of the clinical staging procedure, especially considering the associated—albeit it being minimally invasive—morbidity for the patient as well as cost for its owner. Further studies warrant substantiation—or mitigation—of this observation; until then, the undetermined value of splenic and hepatic fine-needle aspiration as a means of adding to the characterization of a patient’s prognosis may be communicated as such to the client.

The number of cases with hypercalcemia is lower in the present study (11%) than reported in literature (up to 37–52%) [7, 19, 20, 34, 43], and all tested appeared to be of T-cell immunophenotype. A possible explanation could be that almost one-third of the dogs had been pretreated with corticosteroids. Hypercalcemia was not an independent prognostic factor in the present study, as it is associated with T-cell immunophenotype, which is the major prognostic indicator in canine lymphoma [31].

There were not many adverse events during this protocol, except for neutropenia. Some 65% of the dogs had one or multiple neutropenic episodes, frequently leading to postponement of treatment. It has been described that there is a higher risk of neutropenia when L-asparaginase and vincristine are given together [44]. Other studies found that the timing of administration of L-asparaginase was not directly linked to an increased risk [45]. Here we found 24% of the dogs having neutropenia after simultaneous administration of these drugs, however, we also saw 23% of the dogs having neutropenia after single vincristine administration. Therefore, there was no significant increase in neutropenia due to the simultaneous administration and no reason to change the protocol for this adverse event. Wang et al. [46] found that dogs with bone marrow suppression that required dose reductions and treatment delays had longer remissions and lymphoma-specific survival times than those requiring no dose reductions or treatment delays. These findings would indicate that chemotherapy-induced neutropenia may be a useful indicator for predicting tumour responses. In our study, however, we could not find this association.

The present study is subject to several limitations. Firstly, the number of included dogs—although acceptable for a single-institution veterinary study—may still be considered as fairly low. Secondly, the study was designed as a single-arm investigation and possessed no comparative character. Therefore, it remains unclear whether the addition of a lomustine consolidation phase to the mere CHOP-based regime bears true benefit for lymphoma dogs. Future studies—preferentially multi-institutional under inclusion of higher patient numbers- are warranted to address these restrictions and clarify the veritable role of lomustine as well as its most optimal timing within a CHOP-based protocol. Thirdly, the evaluation of lomustine’s potential for compromising hepatic function was not fully evaluable as complete liver enzyme profiles were not available in addition to the bile acid measurements which were performed routinely during the lomustine treatment cycles and revealed no indications of overt liver toxicosis. Finally,and most importantly in the light of recent studies elucidating the significance and varying biologic behaviors of different lymphoma histologic subtypes, the presented study lacked further differentiation of the lymphoma forms beyond the exclusion of low-grade, small cell lymphoma as well as B-and T-cell immunophenotyping. As in human oncology, future veterinary lymphoma investigations are to focus on separately evaluating the varying lymphoma subtypes according to their histologic variants and their immunophenotypic as well as molecular-genetic characteristics. Elucidating their different biologic behavior patterns and responses to therapy sets the path for the long-term goal of identifying subtype-specific if not inter-individual and in this more successful treatment strategies.

## Conclusions

The presented discontinuous combination chemotherapy protocol complemented by a lomustine consolidation phase in dogs with high grade B- and T-cell lymphoma led to comparable response rates and median first remission periods as routine combination chemotherapy protocols. In addition, the treatment was generally well tolerated. The true benefit of lomustine supplementation to a CHOP-based chemotherapy regime must be elucidated in future comparative studies, preferentially focusing on the different histologic types of B- and T-cell lymphoma and taking their varying biologic behaviors and prognoses into consideration.

## Supplementary Information

**Additional file 1.** Clinical characteristics in dogs with lymphoma at diagnosis.

**Additional file 2.** Adverse effects (AE) in 144 dogs treated with multiagent short-term chemotherapy protocol incl. lomustine, graded according to VCOG-CTCAE.

## Data Availability

The datasets used and/or analysed during the current study are available from the corresponding author on reasonable request.

## Abbreviations

CBC: Complete blood count
CHOP: Cyclophosphamide, doxorubicin, vincristine and prednisolone
CR: Complete remission
DFI: Disease free interval
FNA: Fine needle aspiration
HCT: Hematocrit
OS: Overall survival
PD: Progressive disease
PR: Partial remission
SD: Satble disease
VCOG: Veterinary Co-operative Oncology Group

## Footnotes

1 Asparaginase (Asparaginase 5000, -10 000Medac®, Medac, Hamburg, Germany)

2 Vincristine sulfate (cellcristin®, cell pharm GmbH, Bad Vilbel, Germany)

3 Cyclophosphamide (Endoxan®, Baxter Oncology, Frankfurt/Main, Germany)

4 Doxorubicin-HCl (Doxo-cell®, cell pharm GmbH, Bad Vilbel, Germany)

5 Prednisolone (Prednisolon®, Jenapharm, Brehna, Germany)

6 Dexamethasone (Dexamethason®, Jenapharm, Brehna, Germany)

7 SPSS Statistics 26 (IBM SPSS Statistics, IBM Deutschland GmbH, Ehningen, Germany

## Declarations

### Prior publication

Data have been included in a Dissertation “Evaluation zweier Kurzzeit-Chemotherapieprotokolle zur Behandlung des Lymphoms” of Stefanie Limmer at Tierärztliche Hochschule Hannover, but have not been published on-line or elsewhere.
