# Supplementary material for: Efficacy and tolerability of a 12-week combination chemotherapy followed by lomustine consolidation treatment in canine B- and T-cell lymphoma
Source: Acta Vet Scand. 2022 Dec 12;64:36. doi: 10.1186/s13028-022-00660-z (PMC9743771; doi:10.1186/s13028-022-00660-z)
Supplement: Supplementary file 1 — Additional file 1. Clinical characteristics in dogs with lymphoma at diagnosis. [file 13028_2022_660_MOESM1_ESM.pdf]

Additional file 1: Clinical characteristics in dogs with lymphoma at diagnosis

Variable	Distribution	total (n=144)	total (%)	B-cell (n=97)	T-cell (n=34)	median (range)
Age						7,25 years (3-16)
Breeds	Mixed breed	40	28	24	11	
	Golden Retriever	10	7	7	3	
	Bernese Mountain Dog	10	7	8	2	
	German Shepherd	8	6	7		
	Beagle	6	4	3	1	
	Cocker Spaniel	6	4	4	1	
	Rottweiler	5	3	5		
	Hovawart	4	3	2	2	
	other	55	38	37	14	
Sex	male intact	59	41	34	18	
	male castrated	22	15,3	15	6	
	female intact	32	22,2	24	4	
	female spayed	31	21,5	24	6	
Body weight						30,3 kg (5-59)
Neutropenia at diagnosis		3	2	1	2	
Anemia at diagnosis		58	40,3	42	9	
Thrombopenia at diagnosis		32	22,2	15	13	
Hypercalcemia at diagnosis		16	11,2		14	
Anatomical classification	multicentric	123	85,4	94	25	
	alimentary	13	9	3	5	
	Miscellaneous	7	4,9		4	
	mediastinal	1	0,7			
Clinical stage	II	1	0,7			
	III	5	3,5	1	4	
	IV	70	48,6	55	13	
	V	68	47,2	41	17	
Substage	a	61	42,4	50	9	
	b	83	57,6	47	25	
Immunophenotype	B-cell	97	67			
	T-cell	34	24			
	Phenotyping not possible	8	6			
	unclear/vague	5	3			
Clinical signs at diagnosis						21 days (0-80)
	vomiting	13	9	3	6	
	diarrhea	14	10	4	6	
	inappetence	42	29	20	18	
	dyspnea	12	8	10	2	
	weight loss	22	15	11	9	
	lethargy/apathy	41	28	28	10	
	polyuria/polydipsia	15	10	2	11	
Pretreatment with Corticosteroids	yes	47	33	33	11	
