# Supplementary material for: Efficacy and tolerability of a 12-week combination chemotherapy followed by lomustine consolidation treatment in canine B- and T-cell lymphoma
Source: Acta Vet Scand. 2022 Dec 12;64:36. doi: 10.1186/s13028-022-00660-z (PMC9743771; doi:10.1186/s13028-022-00660-z)
Supplement: Supplementary file 2 — Additional file 2. Adverse effects (AE) in 144 dogs treated with multiagent short-term chemotherapy protocol incl. lomustine, graded according to VCOG-CTCAE. [file 13028_2022_660_MOESM2_ESM.pdf]

Additional file 2 Adverse effects (AE) in 144 dogs treated with multiagent short-term chemotherapy protocol incl. lomustine, graded according to VCOG-CTCAE

Toxicosis		Neutropenia		Anemia		Thrombopenia		Lethargy		Anorexia		Diarrhea		Vomiting		Cystitis	
		n	%	n	%	n	%	n	%	n	%	n	%	n	%	n	%
total number of AE		181		855		157		93		111		87		104		13	
affected dogs		85	63	124	91	69	51	63	45	70	51	55	38	60	42	13	9
median number per dog (range)		1 (0-7)		7 (0-15)		1 (0-8)		0 (0-5)		1 (0-1)		0 (0-3)		0 (0-5)		0 (0-1)	
VCOG grade	1	82	45	756	88	99	63	62	67	54	49	62	71	76	73	0	0
	2	55	30	75	9	44	28	26	28	47	42	17	20	24	23	11	85
	3	33	18	19	2	11	7	5	5	9	8	8	9	4	4	2	15
	4	11	6	5	1	3	2	0	0	1	1	0	0	0	0	0	0
	5	-	-	-	-	-	-	0	0	0	0	0	0	0	0	0	0
AE after:	Vincristine/ L-asparaginase	44	24	83	10	9	6	18	19	18	16	19	22	20	19	2	15
	Vincristine	41	23	204	24	5	3	26	28	28	25	17	20	18	17	1	8
	Cyclophosphamid e	38	21	265	31	13	8	17	18	25	23	19	22	36	35	9	69
	Doxorubicin	43	24	268	31	122	78	24	26	33	30	30	34	27	26	1	8
	Lomustine	15	8	35	4	8	5	8	9	7	6	2	2	3	3	0	0

Treatment delay	total		because of neutropenia	
	n	%	n	%
number of treatment delays	173		134	77
affected dogs	83	65	67	52
median number per dog (range)	1 (0-5)		2 (1-5)	
